# Dysregulation of Nociceptin/Orphanin FQ and Dynorphin Systems in the Extended Amygdala of Alcohol Preferring Marchigian Sardinian (msP) Rats

**DOI:** 10.3390/ijms22052448

**Published:** 2021-02-28

**Authors:** Francesca Felicia Caputi, Serena Stopponi, Laura Rullo, Martina Palmisano, Massimo Ubaldi, Sanzio Candeletti, Roberto Ciccocioppo, Patrizia Romualdi

**Affiliations:** 1 Department of Pharmacy and Biotechnology, Alma Mater Studiorum-University of Bologna, Via Irnerio 48, 40126 Bologna, Italy; francesca.caputi3@unibo.it (F.F.C.); laura.rullo3@unibo.it (L.R.); martina.palmisano2@unibo.it (M.P.); sanzio.candeletti@unibo.it (S.C.); 2 Pharmacology Unit, School of Pharmacy, University of Camerino, Madonna delle Carceri, 62032 Camerino, Italy; serena.stopponi@unicam.it (S.S.); massimo.ubaldi@unicam.it (M.U.)

**Keywords:** addiction, ethanol, nociceptin, dynorphin, BDNF, CRF, NOP, CRF-R1, KOP, Trk-B

## Abstract

Previous studies have shown that genetically selected Marchigian Sardinian alcohol-preferring (msP) rats consume excessive amounts of ethanol to self-medicate from negative moods and to relieve innate hypersensitivity to stress. This phenotype resembling a subset of alcohol use disorder (AUD) patients, appears to be linked to a dysregulation of the equilibrium between stress and antistress mechanisms in the extended amygdala. Here, comparing water and alcohol exposed msP and Wistar rats we evaluate the transcript expression of the anti-stress opioid-like peptide nociceptin/orphanin FQ (N/OFQ) and its receptor NOP as well as of dynorphin (DYN) and its cognate κ-opioid receptor (KOP). In addition, we measured the transcript levels of corticotropin-releasing factor (CRF), CRF receptor 1 (CRF1R), brain-derived neurotrophic factor (BDNF) and of the tropomyosin receptor kinase B receptor (Trk-B). Results showed an innately up-regulation of the CRFergic system, mediating negative mood and stress responses, as well as an inherent up-regulation of the anti-stress N/OFQ system, both in the amygdala (AMY) and bed nucleus of the stria terminalis (BNST) of msP rats. The up-regulation of this latter system may reflect an attempt to buffer the negative condition elicited by the hyperactivity of pro-stress mechanisms since results showed that voluntary alcohol consumption dampened N/OFQ. Alcohol exposure also reduced the expression of dynorphin and CRF transmissions in the AMY of msP rats. In the BNST, alcohol intake led to a more complex reorganization of these systems increasing receptor transcripts in msP rats, along with an increase of CRF and a decrease of N/OFQ transcripts, respectively. Moreover, mimicking the effects of alcohol in the AMY we observed that the activation of NOP receptor by intracerebroventricular administration of N/OFQ in msP rats caused an increase of BDNF and a decrease of CRF transcripts. Our study indicates that both stress and anti-stress mechanisms are dysregulated in the extended AMY of msP rats. The voluntary alcohol drinking, as well as NOP agonism, have a significant impact on neuropeptidergic systems arrangement, bringing the systems back to normalization.

## 1. Introduction

Addictive drugs, including alcohol, are abused by humans and, under some experimental condition, are voluntarily consumed also by rodents [1]. Therefore, specific molecular determinants of excessive alcohol consumption and the neurobiology of alcoholism, not easily examinable in humans, can be analyzed in animal models. In this regard, the use of genetically selected alcohol-preferring animals represents a suitable research tool to explore the molecular complexities underlying genetic predisposition to excessive ethanol (EtOH) drinking.

Marchigian Sardinian alcohol-preferring (msP) rats have been genetically selected for high 10% EtOH preference; they exhibit an innate propensity to excessive drinking, with an anxious phenotype that ameliorates following EtOH consumption [2,3]. Based on these findings, it has been proposed that genetic selection in msP rats co-segregates with the expression of a highly anxious and stress vulnerable phenotype resembling a specific subpopulation of alcoholic patients that attempt to drink to self-medicate from negative mood [4,5]. In this regard, it is well known that alcohol use disorder (AUD) shows high comorbidity with mood disorders [6,7]. Under this condition, drinking is preferentially motivated by negative rather than positive reinforcement mechanisms [8,9,10].

On that note, the hypothesis has been formulated that specific neurochemical circuits and molecular mechanisms are engaged in the rewarding properties of alcohol [11,12,13,14,15,16], as well as in alcohol consumption for self-medication purposes [3,17]. Based on this conceptualization, the aim of this study was to investigate the molecular basis for differential vulnerability to AUD by analyzing the expression of neurotrophic and stress-related factors in msP compared to Wistar rats. In particular, we evaluated whether msP rats might have innate dysregulation in the expression and function of opioid neuropeptidergic systems linked to negative reinforcement and the modulation of negative mood, such as the pro-stress dynorphin (DYN)/κ-opioid receptor (KOP) and the anti-stress nociceptin/orphanin FQ (N/OFQ)–NOP receptor (NOP) systems. Moreover, we decided to expand our analysis to the corticotropin-releasing factor (CRF)–CRF receptor 1 (CRF1R) as it is tightly linked to the regulation of the above mentioned opioidergic mechanisms. In fact, it is well known that both CRFergic and DYNergic transmissions mediate stress response and contribute to the expression of negative reinforcement [18,19,20,21]. Whereas, activation of NOP by N/OFQ results in a functional CRF antagonism and mediates anti-stress responses [19,22,23,24,25]. Finally, we analyzed the expression of the brain-derived neurotrophic factor (BDNF)- tropomyosin receptor kinase B receptor (Trk-B) system as BDNF regulates ethanol intake by activation of downstream gene products like pDYN [26] and is known to have a role in alcohol dependence vulnerability [25,27,28,29]. In this regard, some evidence indicated that animals characterized by low BDNF expression in several brain regions, including amygdala (AMY), display anxiety-like behaviors together with higher alcohol preference [30]. In addition, other studies demonstrated the BDNF contribution to drug craving, seeking and relapse [31,32], thus highlighting the involvement of BDNF in multiple facets of addiction [33].

We focused our attention to the AMY and the bed nucleus of the stria terminalis (BNST), as these regions represent important crossroads where mechanisms regulating reward, stress and addiction intersect each other [13,14,15,16,17,18,19,20,21,22,23,24,25,26,27,28,29,30,31,32,33,34]. Besides the analysis of basal gene expression for the above mentioned systems, we also evaluated how the chronic intermittent consumption of alcohol (CIE), a well characterized animal model of binge drinking in humans [35,36,37], may impact on the expression of the investigated neuropeptidergic systems in msP or Wistar rats. Finally, stemming from the hypothesis that N/OFQ counters the actions of CRF and DYN in stress response regulation, and that activation of NOP reduces alcohol drinking in msP rats by blunting the activity of these systems [23], we evaluated how activation or blockade of NOP receptor would impact the gene expression of these stress-related systems in the AMY of msP rats. 

## 2. Results

### 2.1. Voluntary 10% EtOH Intake 

**Figure 1:**
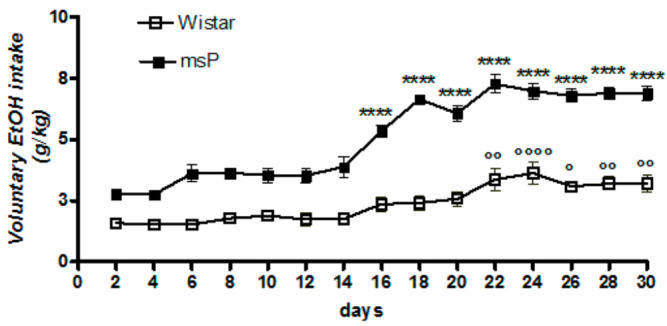
Daily consumption of 10% (*v*/*v*) alcohol assessed by the two-bottle free choice drinking paradigm. Values are expressed as mean ± SEM of alcohol intake (g/kg) measured for Wistars (° *p* < 0.05, °° *p* < 0.01 and °°°° *p* < 0.0001) or msP rats (**** *p* < 0.0001) vs. their respective first drinking session); ANOVA followed by Tukey’s multiple comparison test.

Overall ANOVA revealed a significant effect of strain [F_(1,330)_ = 689.5, *p* < 0.0001], time [F_(14,330)_ = 37.55, *p* < 0.0001] and strain × time interaction [F_(14,330)_ = 6.955, *p* < 0.0001], highlighting a significantly different pattern of drinking between msP and Wistar rats across the 30-day of intermittent EtOH exposure (Figure 1). Tukey’s multiple comparison *post hoc* analysis revealed a significant escalation of alcohol consumption in msP rats (Figure 1). 

### 2.2. Gene Expression Analysis in the AMY of Water Controls and 10% EtOH Exposed Wistar and msP Rats 

**Figure 2:**
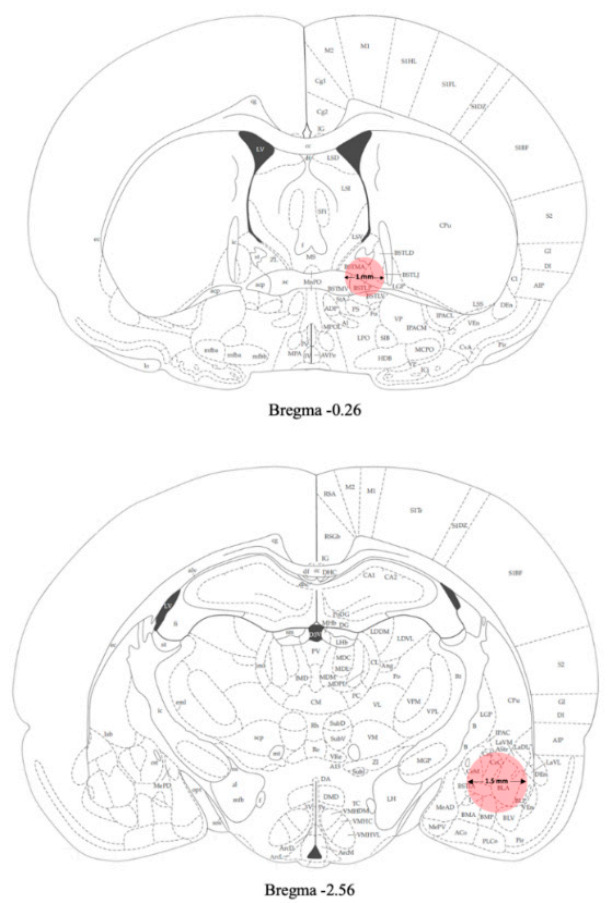
Schematic representation of the areas (pink) punched for gene expression analyses [38] (see Materials and Methods section for details).

Samples of amygdala were punched from the brain and used for gene expression analyses (Figure 2).

#### 2.2.1. pN/OFQ Expression

**Figure 3:**
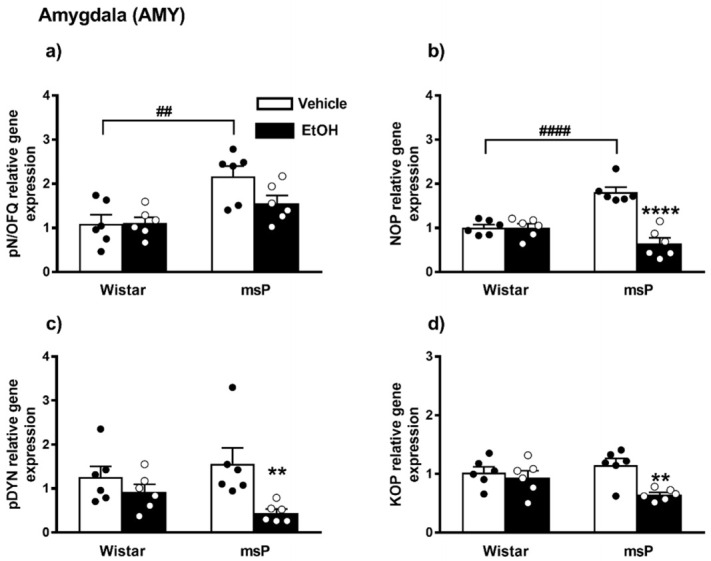
mRNA levels of pN/OFQ, NOP, pDYN and KOP (**a**–**d**) in the AMY of Wistar and msP rats after Vehicle or chronic intermittent EtOH (CIE) consumption. Data represent 2^−DDCt^ values calculated by DDCt method and are expressed as mean ± SEM of six rats per group (** *p* < 0.01 and **** *p* < 0.0001 vs. their respective Vehicle; ^##^ *p* < 0.01 and ^####^ *p* < 0.0001 vs. Wistar Vehicle). See single values reported as dots in each group and Data Analysis for details.

Overall ANOVA revealed a significant effect of strain on the pN/OFQ gene expression [F_(1,20)_ = 16.17, *p* = 0.0007]; no significant effect of EtOH drinking [F_(1,20)_ = 2.510, *p* = 0.1288, n.s.] and of strain × EtOH drinking interaction [F_(1,20)_ = 2.731, *p* = 0.1140, n.s.] were instead observed. Sidak’s multiple comparison *post hoc* tests revealed that the basal pN/OFQ mRNA levels were significantly higher in msP rats compared to Wistars (msP Vehicle = 2.17 ± 0.23 vs. Wistar Vehicle = 1.00 ± 0.20, *p* = 0.0014) (Figure 3a). A trend of decrease in pN/OFQ mRNA levels was induced by CIE consumption in msP rats only (msP EtOH = 1.56 ± 0.18 vs. msP Vehicle = 2.17 ± 0.23, *p* = 0.0651) (Figure 3a).

#### 2.2.2. NOP Expression

ANOVA indicated a significant effect of strain [F_(1,20)_ = 4.680, *p* = 0.0428] and EtOH drinking [F_(1,20)_ = 32.96, *p* < 0.0001]. There was also a significant strain x EtOH drinking interaction effect [F_(1,20)_ = 32.47, *p* < 0.0001]. As shown by post hoc analysis a highly significant difference between Wistar and msP rats was observed in the innate levels of NOP mRNA that was higher in msPs compared to Wistars a (msP Vehicle = 1.81 ± 0.11 vs. Wistar Vehicle = 1.00 ± 0.07, *p* < 0.0001) (Figure 3b). A significant NOP down-regulation was detected after CIE consumption in msP rats only (msP EtOH = 0.64 ± 0.13 vs. msP Vehicle = 1.81 ± 0.11, *p* < 0.0001) (Figure 3b). 

#### 2.2.3. pDYN Expression

ANOVA indicated a significant effect of CIE on pDYN gene expression [F_(1,20)_ = 9.319, *p* = 0.0063]. No significant effect of strain [F_(1,20)_ = 0.1264, *p* = 0.7260, n.s.] and of strain x EtOH drinking interaction [F_(1,20)_ = 2.728, *p* = 0.1142, n.s.] were detected. Sidak’s multiple comparison *post hoc* tests revealed no significant differences in the pDYN gene expression basal levels between Wistar and msP rats (msP Vehicle = 1.56 ± 0.35 vs. Wistar Vehicle = 1.00 ± 0.25, n.s.) (Figure 3c). A significant down-regulation of pDYN gene expression was detected after CIE consumption in msP rats only (msP EtOH = 0.44 ± 0.08 vs. msP Vehicle = 1.56 ± 0.35, *p* = 0.0067) (Figure 3c).

#### 2.2.4. KOP Expression

ANOVA of KOP gene expression indicated a significant effect of EtOH drinking [F_(1,20)_ = 9.367, *p* = 0.0062], and also a significant effect of strain x EtOH drinking interaction [F_(1,20)_ = 4.869, *p* = 0.0392]. No significant effect of strain [F_(1,20)_ = 0.7673, *p* = 0.3914, n.s.] was reported. Sidak’s multiple comparisons revealed no significant differences in the basal KOP mRNA levels between Wistar and msP rats (msP Vehicle = 1.15 ± 0.11 vs. Wistar Vehicle = 1.00 ± 0.09, n.s.) (Figure 3d). A significant down-regulation of KOP gene expression was detected after CIE consumption in msP rats only (msP EtOH = 0.64 ± 0.04 vs. msP Vehicle = 1.15 ± 0.11, *p* = 0.0027) (Figure 3d).

#### 2.2.5. CRF Expression

**Figure 4:**
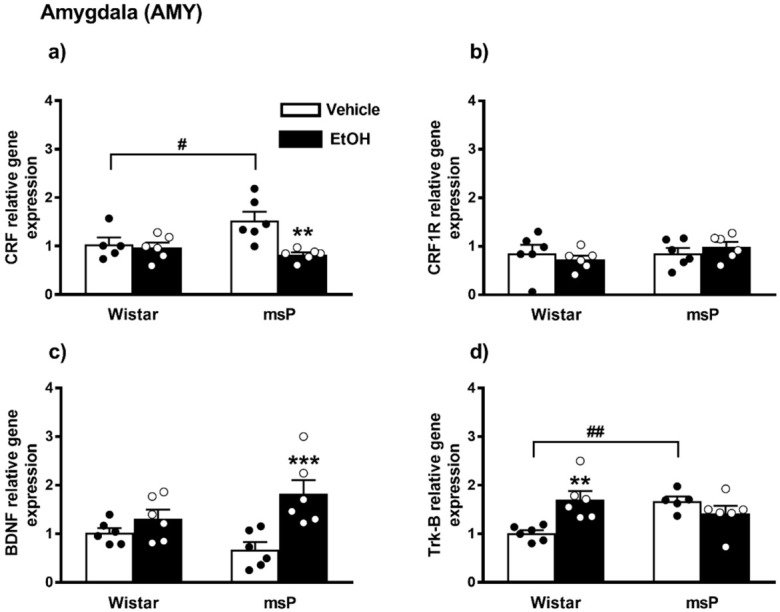
mRNA levels of CRF, CRF1R, BDNF and Trk-B (**a**–**d**) in the AMY of Wistar and msP rats after Vehicle or chronic intermittent EtOH consumption paradigm. Data represent 2^−DDCt^ values calculated by DDCt method and are expressed as mean ± SEM of five/six rats per group (** *p* < 0.01 and *** *p* < 0.001 vs. their respective Vehicle; ^#^ *p* < 0.05 and ^##^ *p* < 0.01 vs. Wistar Vehicle). One outlier ((**a**) Wistar vehicle group) and one outlier ((**d**) msP vehicle group) were not included in data analysis.

ANOVA revealed a significant effect of EtOH drinking on the CRF gene expression [F_(1,19)_ = 9.307, *p* = 0.0066], and also a significant strain x EtOH drinking interaction [F_(1,19)_ = 6.408, *p* = 0.0203]. No significant effect of strain [F_(1,19)_ = 1.869, *p* = 0.1875, n.s.] was observed. Sidak’s multiple comparisons revealed significant differences in the basal levels of CRF mRNA that were up-regulated in the msP rats compared to Wistars (msP Vehicle = 1.53 ± 0.18 vs. Wistar Vehicle = 1.00 ± 0.14, *p* = 0.0286) (Figure 3a). A significant CRF gene expression down-regulation was detected after CIE consumption in msP rats only (msP EtOH = 0.82 ± 0.05 vs. msP Vehicle = 1.53 ± 0.18, *p* = 0.0014) (Figure 4a).

#### 2.2.6. CRF1R Expression

ANOVA indicated no significant effect of strain [F_(1,20)_ = 1.056, *p* = 0.3165, n.s.], EtOH drinking [F_(1,20)_ = 0.0005, *p* = 0.9817, n.s.] and of strain x EtOH drinking interaction [F_(1,20)_ = 1.156, *p* = 0.2951] on CRF1R gene expression in the AMY (Figure 4b).

#### 2.2.7. BDNF Expression

ANOVA indicated a significant effect of EtOH drinking on the BDNF gene expression [F_(1,20)_ = 14.43, *p* = 0.0011], and also a significant strain x EtOH drinking interaction [F_(1,20)_ = 5.048, *p* = 0.0361]. No significant effect of strain [F_(1,20)_ = 0.1823, *p* = 0.6740, n.s.] was observed. The Sidak’s multiple comparison *post hoc* test revealed no significant differences in the BDNF basal levels between msP and Wistars rats (msP Vehicle = 0.68 ± 0.15 vs. Wistar Vehicle = 1.00 ± 0.09, n.s) (Figure 4c). CIE induced a significant up-regulation of BDNF gene expression in msP rats only (msP EtOH = 1.82 ± 0.28 vs. msP Vehicle = 0.68 ± 0.15, *p* = 0.0007) (Figure 4c).

#### 2.2.8. Trk-B Expression

ANOVA indicated no significant effect of strain [F_(1,19)_ = 1.947, *p* = 0.1791, n.s.] and of EtOH drinking [F_(1,19)_ = 2.643, *p* = 0.1205, n.s.] on the Trk-B gene expression. However, a significant strain x EtOH drinking interaction was observed [F_(1,19)_ = 12.42, *p* = 0.0023]. Sidak’s multiple comparison tests revealed that the Trk-B mRNA basal level was significantly higher in the msP rats compared to Wistars (msP Vehicle = 1.47 ± 0.22 vs. Wistar Vehicle = 1.00 ± 0.06, *p* = 0.0060) (Figure 4d). Multiple comparison tests revealed also a significant increase of Trk-B levels after CIE consumption in Wistar rat only (Wistar EtOH = 1.70 ± 0.17 vs. Wistar Vehicle = 1.00 ± 0.06, *p* = 0.0028) (Figure 4d). Results are schematically represented in Table 1.

### 2.3. Gene Expression Analysis in the BNST of Water Controls and 10% EtOH Exposed Wistar and msP Rats 

Samples of BNST were punched from the brain and used for gene expression analyses (Figure 2).

#### 2.3.1. pN/OFQ Expression

**Figure 5:**
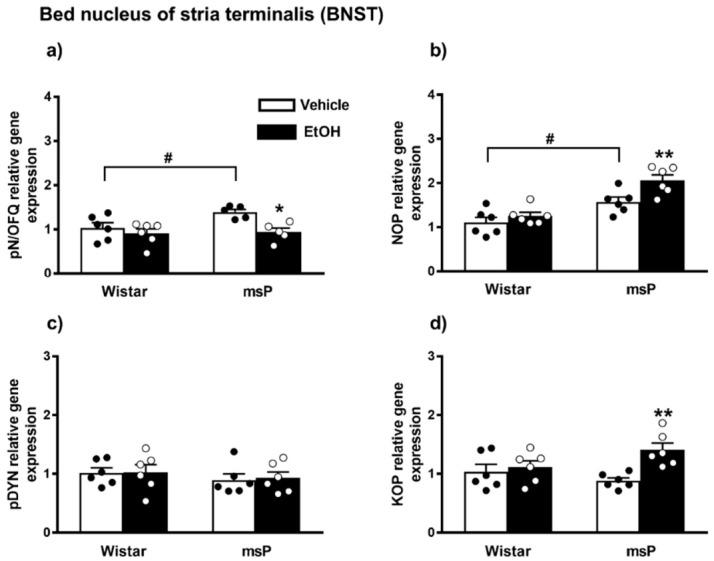
mRNA levels of pN/OFQ, NOP, pDYN and KOP (**a**–**d**) in the BNST of Wistar and msP rats after Vehicle or chronic intermittent EtOH consumption. Data represent 2^−DDCt^ values calculated by DDCt method and are expressed as mean ± SEM of five/six rats per group (* *p* < 0.05 and ** *p* < 0.01 vs. their respective Vehicle; ^#^ *p* < 0.01 vs. Wistar Vehicle). One outlier ((**a**) msP vehicle group) and one outlier ((**a**) mSP EtOH group) were not included in data analysis.

ANOVA indicated a significant effect of EtOH drinking [F_(1,18)_ = 10.85, *p* = 0.0040] and of strain [F_(1,18)_ = 5.534, *p* = 0.0302] on the pN/OFQ gene expression. No significant effect of strain x EtOH drinking interaction [F_(1,18)_ = 1.405, *p* = 0.2513, n.s.] was instead observed. The Sidak’s multiple comparison *post hoc* test revealed that basal pN/OFQ mRNA levels were significantly higher in msP rats compared to Wistars (msP Vehicle = 1.39 ± 0.06 vs. Wistar Vehicle = 1.00 ± 0.11, *p* = 0.0440) (Figure 5a). A significant down-regulation of pN/OFQ mRNA levels was detected after CIE consumption in msP rats only (msP EtOH = 0.94 ± 0.09 vs. msP Vehicle = 1.39 ± 0.06, *p* = 0.0143) (Figure 5a).

#### 2.3.2. NOP Expression

ANOVA showed a significant effect of strain [F_(1,20)_ = 33.90, *p* < 0.0001] and EtOH drinking [F_(1,20)_ = 8.560, *p* = 0.0084] on the NOP mRNA levels. No significant effect of strain x EtOH drinking interaction [F_(1,20)_ = 2.370, *p* = 0.1394, n.s.] was observed. The msP line exhibited higher NOP mRNA basal levels than Wistars (msP Vehicle = 1.57 ± 0.11 vs. Wistar Vehicle = 1.00 ± 0.12, *p* = 0.0132) (Figure 4b). A significant NOP up-regulation was detected after CIE consumption in msP rats only (msP EtOH = 2.06 ± 0.12 vs. msP Vehicle = 1.57 ± 0.11, *p* = 0.0099) (Figure 5b). 

#### 2.3.3. pDYN Expression

ANOVA indicated no significant effect of strain [F_(1,20)_ = 1.043, *p* = 0.3194, n.s.], EtOH drinking [F_(1,20)_ = 0.045, *p* = 0.8337, n.s.] and strain x EtOH drinking interaction [F_(1,20)_ = 0.018, *p* = 0.8948, n.s.] on the pDYN gene expression in the BNST (Figure 5c). 

#### 2.3.4. KOP Expression

ANOVA showed a significant effect of EtOH drinking [F_(1,20)_ = 8.559, *p* = 0.0084] and a significant effect of strain x EtOH drinking interaction [F_(1,20)_ = 4.655, *p* < 0.0433]. No significant effect of strain [F_(1,20)_ = 0.4438, *p* = 0.5129, n.s.] was observed. Sidak’s multiple comparison *post hoc* tests revealed no significant differences in KOP mRNA basal levels between Wistar and msP rats (msP Vehicle = 0.88 ± 0.04 vs. Wistar Vehicle = 1.00 ± 0.10, n.s.) (Figure 5d). A significant up-regulation of KOP gene expression was detected after CIE consumption in msP rats only (msP EtOH = 1.41 ± 0.09 vs. msP Vehicle = 0.88 ± 0.04, *p* = 0.0036) (Figure 5d).

#### 2.3.5. CRF Expression

**Figure 6:**
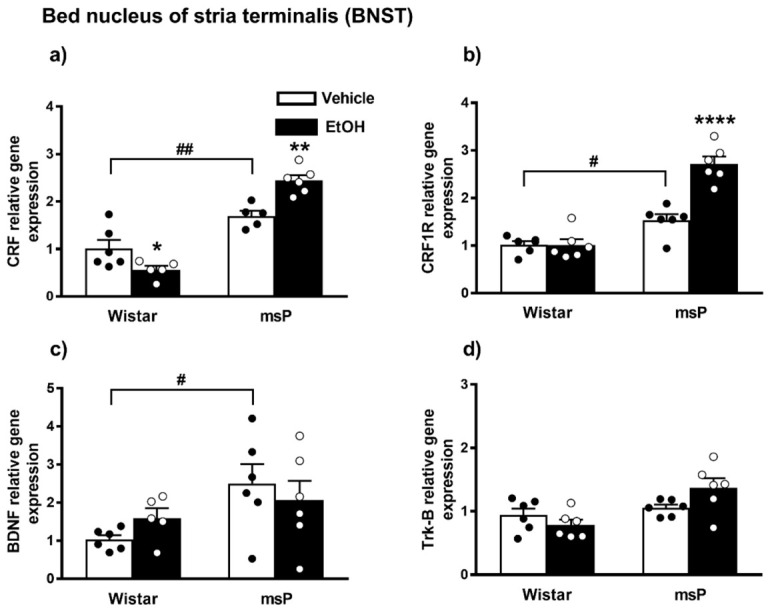
mRNA levels of CRF, CRF1R, BDNF and Trk-B (**a**–**d**) in the BNST of Wistar and msP rats after Vehicle or chronic intermittent EtOH consumption. Data represent 2^−DDCt^ values calculated by DDCt method and are expressed as mean ± SEM of five/six rats per group (* *p* < 0.05, ** *p* < 0.01 and **** *p* < 0.0001 vs. their respective Vehicle; ^#^ *p* < 0.05 and ^##^ *p* < 0.01 vs. Wistar Vehicle). Three outliers ((**a**) Wistar EtOH group, msP vehicle group; out-panel c, Wistar EtOH group) were not included in data analysis.

ANOVA displayed a significant effect of strain [F_(1,18)_ = 96.15, *p* < 0.0001] and strain x EtOH drinking interaction [F_(1,18)_ = 20.92, *p* = 0.0002] on the CRF gene expression. No significant effect of EtOH drinking [F_(1,18)_ = 1.258, *p* = 0.2767, n.s.] was observed. The Sidak’s multiple comparison *post hoc* test revealed that the basal CRF mRNA levels were significantly higher in msP rats compared to Wistars (msP Vehicle = 1.70 ± 0.10 vs. Wistar Vehicle = 1.00 ± 0.19, *p* = 0.0033) (Figure 6a). A significant down-regulation in the CRF mRNA levels was detected after CIE consumption in Wistar rats (Wistar EtOH = 0.56 ± 0.08 vs. Wistar Vehicle = 1.00 ± 0.19, *p* = 0.00498) (Figure 6a), whereas the same EtOH exposure evoked a significant increase of CRF gene expression levels in msPs (msP EtOH = 2.44 ± 0.11 vs. msP Vehicle = 1.70 ± 0.10, *p* = 0.0016) (Figure 6a).

#### 2.3.6. CRF1R Expression

ANOVA analysis indicated a significant effect of strain [F_(1,20)_ = 79.24, *p* < 0.0001], EtOH drinking [F_(1,20)_ = 22.61, *p* = 0.0001] and also a significant effect of strain × EtOH drinking interaction [F_(1,20)_ = 22.79, *p* < 0.0001] on CRF1R expression. *Post hoc* test revealed higher CRF-R1 mRNA basal levels in the msP rats compared to Wistar animals (msP Vehicle = 1.53 ± 0.13 vs. Wistar Vehicle = 1.00 ± 0.07, *p* = 0.0169) (Figure 5b). A significant CRF-R1 up-regulation was detected after CIE consumption in msP rats only (msP EtOH = 2.71 ± 0.16 vs. msP Vehicle = 1.53 ± 0.13, *p* < 0.0001) (Figure 6b).

#### 2.3.7. BDNF Expression

ANOVA indicated a significant effect of strain [F_(1,19)_ = 5.895, *p* = 0.0253] on the BDNF gene expression in the BNST brain region. No significant effect of EtOH drinking [F_(1,19)_ = 0.024, *p* = 0.8794, n.s.] and of strain x EtOH drinking interaction [F_(1,19)_ = 1.560, *p* = 0.2269, n.s.] was observed. The Sidak’s multiple comparison *post hoc* test revealed no significant differences in the BDNF mRNA levels after CIE consumption in both Wistar and msP rat strain (Wistar EtOH = 1.59 ± 0.26 vs. Wistar Vehicle = 1.00 ± 0.11, n.s.; msP EtOH = 2.06 ± 0.51 vs. msP Vehicle = 2.50 ± 0.51 n.s.) (Figure 6c), but disclosed a significant basal BDNF gene expression up-regulation in msP rats compared to Wistar (msP Vehicle = 2.50 ± 0.51 vs. Wistar Vehicle = 1.00 ± 0.11, *p* = 0.0304) (Figure 6c).

#### 2.3.8. Trk-B Expression

ANOVA of Trk-B gene expression in the BNST indicated a significant effect of strain [F_(1,20)_ = 10.90, *p* = 0.0036] and strain x EtOH drinking interaction [F_(1,20)_ = 5.007, *p* = 0.0368]. No significant effect of EtOH drinking [F_(1,20)_ = 0.5776, *p* = 0.4561, n.s.] was observed. Sidak’s multiple comparison *post hoc* tests revealed no significant differences in the basal mRNA level of Trk-B between Wistar and msP rats (msP Vehicle = 1.05 ± 0.05 vs. Wistar Vehicle = 1.00 ± 0.10, n.s.) (Figure 6d). Results are schematically represented in Table 1.

### 2.4. Effect of Intracerebroventricular Injection of N/OFQ or UFP101 on Neuropeptide Gene Expression in the Amygdala of msP Rats 

**Figure 7:**
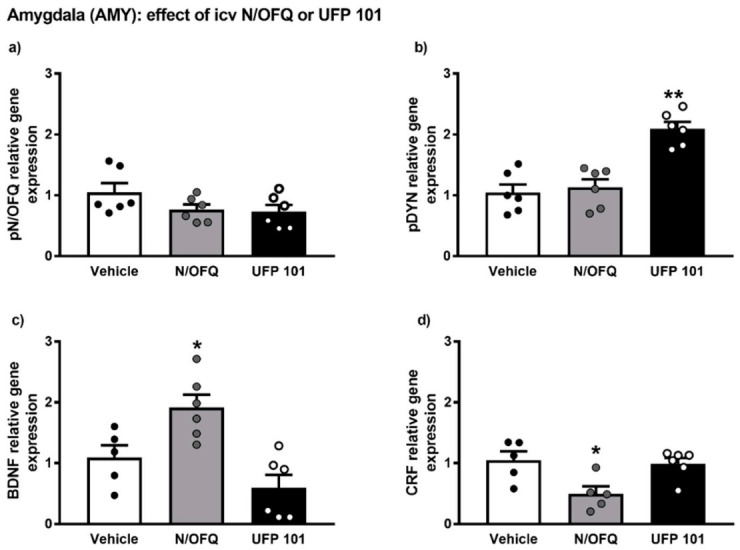
mRNA levels of pN/OFQ, pDYN, BDNF and CRF (**a**–**d**) in the amygdala (AMY) of msP rats after icv injection of N/OFQ or UFP 101. Both the drugs were dissolved in sterile isotonic saline and were injected icv in a volume of 1 µL/rat. Data represent 2^−DDCt^ values calculated by DDCt method and are expressed as mean ± standard error of the mean (SEM) of five/six rats per group (* *p* < 0.05, ** *p* < 0.01 vs. Vehicle). One outlier ((**c**) Vehicle group), one outlier ((**d**) Vehicle group) and one outlier ((**d**) N/OFQ group) were not included in data analysis.

The gene expression alterations observed after icv injection of N/OFQ or UFP 101 are presented in Figure 7.

Overall ANOVA analysis indicated no significant effect of N/OFQ or UFP 101 administration on pN/OFQ gene expression in the AMY of msP rats. On the contrary, a significant up-regulation of pDYN mRNA levels was detected following icv injection of UFP 101 (UFP 101 treated-group = 2.09 ± 0.11 vs. msP Vehicle = 1.00 ± 0.13, *p* < 0.01) (Figure 7b). 

ANOVA revealed that icv administration of N/OFQ evoked a significant up-regulation of BDNF gene expression (N/OFQ treated-group = 1.91 ± 0.21 vs. msP Vehicle = 1.00 ± 0.20, *p* < 0.05) (Figure 7c) and a down-regulation of CRF mRNA levels (N/OFQ treated-group = 0.49 ± 0.11 vs. msP Vehicle = 1.00 ± 0.15, *p* < 0.05) (Figure 6d) in the AMY of msP rats.

## 3. Discussion 

The major finding of our study is that msP and Wistar rats have a different organization of AMY and BNST neurochemical circuits involved in the regulation of stress and alcohol drinking. Moreover, we found that CIE has different impact on the neuropeptidergic regulation of AMY and BNST in msP compared to Wistar rats. Specifically, we found that compared to Wistars, msP rats exhibited about two-fold higher innate levels of the opioid-like peptide transcripts pN/OFQ and of its cognate NOP receptor in the AMY. These results are in agreement with previous studies using in situ hybridization and autoradiography techniques in which it was found that compared to Wistars msP rats have higher pN/OFQ and NOP receptor expression levels in this area [39]. Here, we extended this observation and showed that CIE drinking led to a significant down-regulation of NOP (a trend toward reduction of pN/OFQ was also detected) in msP rats but not in Wistars. Higher innate expression levels of pN/OFQ and NOP mRNA were also detected in the BNST of msP rats compared to Wistars. However, different from what occurred in the AMY, in the BNST of msP rats CIE up-regulated the gene expression of NOP and reduced that of pN/OFQ.

When we analyzed the dynorphinergic system, no basal differences between Wistar and msP rats in the innate levels of pDYN and KOP mRNAs in the AMY or in the BNST were detected. Consistent with earlier studies, we also found that in the AMY and BNST msP rats show a general overexpression of the CRF-CRF1R system [5,40,41]. It is known that in msP rats voluntary EtOH drinking normalized this overexpression, bringing it down to the levels observed in Wistars [40]. Here we replicated this observation, however we also found that, in contrast to what observed in the AMY, ethanol drinking further enhanced the expression of CRF and CRF1R transcripts in the BNST of msP rats. 

When we analyzed BDNF mRNA, we found no significant differences between msP and Wistar rat in the AMY. Conversely, we found higher innate levels of Trk-B mRNA in the msP animal compared to Wistar rat in this region. CIE selectively enhanced BDNF expression in msP rats and increased Trk-B gene expression in Wistar rats, thus suggesting differential effects of ethanol in these two rat lines. In the BNST, innate BDNF levels were higher in the msP rats compared to the Wistars. 

Globally, these data suggest a different modulation of BDNF transmission in the AMY and in the BNST of msPs, compared to Wistar rats. In this respect, it is worth noticing that ethanol drinking selectively increased BDNF levels in the AMY of msP rats without affecting it in the BNST. It is tempting to hypothesize that the dysregulation of BDNF contributes to the high alcohol and anxious drinking phenotype of msP rats, and similarly to what was observed for CRF, EtOH consumption could be motivated by the attempt to rebalance the activity of this neurotrophic system. This hypothesis is supported by earlier studies showing that the BDNF pathway is affected by various drugs of abuse [42], it is involved in the expression of anxiety-like behaviors and is strongly related to excessive alcohol-drinking and relapse [31,43,44]. In particular, it has been demonstrated that the reduction of BDNF expression promotes a greater preference for alcohol consumption [29,45,46,47]. 

Altogether, these findings indicate that msP and Wistar rats are characterized by profound innate differences in the expression of a number of neuronal systems in brain areas involved in the regulation of stress and excessive EtOH drinking. CIE tends to normalize some of these differences, while exacerbates others. 

Earlier work showed that compared to Wistars msP rats show upregulation of several genes associated with alcohol metabolism [3]. This may lead to faster alcohol metabolism that in turn can contribute to the expression of the high drinking phenotype of msP rats. However it is also known that voluntary alcohol intake of msP rats leads to blood ethanol concentrations (BECS) up to 70–80 mg/dL [3]. Whereas voluntary drinking in Wistars being lower leads to BECS are around 10–25 mg/dL [48,49]. This suggests that in msP rats the high propensity to drink elevated amounts of alcohol cannot be simply attributed to its faster elimination. This further supporting the notion that the high drinking motivation of msPs is linked to the attempt to experience the pharmacological effects of the alcohol. 

Present data are not sufficient to understand the exact significance of single neuropeptidergic system differences and how each one of these neuropeptides may impact on the expression of a highly alcohol drinking and vulnerable phenotype. However, analyzing them at global level it is possible to observe that neurotransmitter systems that contribute to mediate negative mood and stress responses like CRF are generally up-regulated in rats with innate predisposition to excessive EtOH drinking. On the other hand, upregulation of the opioid-like N/OFQ system that acts as an anti-stress system and mediates anxiolytic actions is also observed. Changes in N/OFQ transmission towards its upregulation may reflect a physiological mechanism aimed at compensating for the negative mood and stress elicited by enhanced pro-stress mechanisms [24,50,51]. Alcohol, due to its anxiolytic effects, may bring the N/OFQ systems back to normalization because its up-regulation is no longer needed to counteract the innate negative emotional state that characterizes msP rats. This hypothesis is corroborated by data showing that drinking, not only reduced the expression of the N/OFQ but, at least in the AMY also lowered the levels of DYN and CRF transcripts, suggesting a global normalization of stress and anti-stress systems in this region. 

In the present study, to allow the precise monitoring of drinking rats were single housed. Rats are social animals and isolation could influence the response to alcohol drinking. However, a recent work suggested that, over 30 days of free access to alcohol, no significant alcohol intake differences between pair and individually housed rats have been found [52]. Most important, in our experiments the water control group was also single housed, thus gene expression differences among groups cannot be attributed to the isolation *per se*. 

A potential limitation of our work is that, given the method used to collect the brain nuclei a slight portion of the anterior commissure has been sampled together with the BNST. However, since this portion is very limited we assume that the gene expression levels detected in this brain structure is mostly attributable to the BNST. We should also point out that our sampling procedure was not intended at collecting subregions of the BNST or the AMY hence we cannot infer information on gene expression changes occurring in specific subnuclei of these highly heterogeneous areas. It should be mentioned, however, that for the AMY the tissue samples that we collected contained mostly the central nucleus and basolateral subregions but not the medial amygdaloid nucleus. Moreover, sampling was directed to the rostral portion of AMY because it is involved in processing alcohol-related cues, is where the extended amygdala originates, it is contiguous to the BNST and is rich of opioid receptors and peptides [53,54,55]. 

Previous work showed N/OFQ and NOP receptor agonists decreased alcohol consumption [39,56,57], ethanol-induced conditioned place preference [58] and cue-induced relapse to alcohol drinking [59]. Moreover the N/OFQ system plays a central role in the control of stress response [60] and several evidence demonstrated that activation of NOP by central administration N/OFQ induced anxiolytic-like effects across several experimental models [60,61,62,63,64]. Recent evidence showed that also NOP antagonists are effective in reducing alcohol drinking [65,66]. Whereas blockade of the NOP receptor attenuates anxiety-like responses associated with stress or to depressive-like condition [67].

Based on these findings, it was tempting to hypothesize that mimicking the effect of EtOH, exogenous administration of NOP agonists and antagonists would affect the expression of pro-stress systems in the same directions. To evaluate this possibility, we investigated the effect of icv injection of N/OFQ and UFP101 on N/OFQ, DYN, CRF and BDNF transcripts in the AMY. Overlapping the effects of EtOH drinking, injection of N/OFQ reduced the expression of CRF and increased that of BDNF. On the other hand, DYN levels did not show significant changes. We speculate that acute icv injection of N/OFQ, differently from chronic alcohol intake, did not change the expression of DYN because a single administration was not sufficient to activate the molecular machinery responsible for the downregulation of DYN gene expression. Noteworthy, earlier work demonstrated that alcohol drinking reduction is more pronounced following chronic administration rather than an acute injection of N/OFQ [59]. We also found that after administration of the NOP antagonist UFP101 the only gene affected was DYN that markedly increased. First of all, this finding indicates that endogenous N/OFQ exerts a tonic negative control over DYN transmission. Secondly, it suggests that NOP agonism and antagonism may regulate EtOH drinking through distinct mechanisms.

As mentioned above, CRF gene expression decreased in the AMY after CIE, but unexpectedly both CRF and CRF1 transcripts were increased in the BNST. This region has a highly complex structure and it is known to be involved in the control of both anxiogenic and anxiolytic pathways [68]. The BNST receives CRF fibers which predominantly come from the central nucleus (CeA) of the AMY [68], but also includes CRF-producing neurons [68,69]. It is conceivable that enhanced expression of CRF1R gene in the BNST is a mechanism aimed at compensating alcohol drinking-induced decrease of CRF transmission from the AMY. This is consistent with the observed reduction in CRF gene expression in this latter region in msP rats taking alcohol. Whereas, the increase of BNST CRF gene expression is probably occurring in those CRF cells belonging to local BNST circuitries, that may actually work as anxiety-stop signals [68]. The disentanglement of the significance of these gene expression changes at neurocircuit levels is not possible through the technical approach used here. Future studies will have to be carried out to confirm our hypothesis. 

## 4. Materials and Methods

### 4.1. Animals 

Male Wistar and msP rats, weighing approximately 250–300 g at the beginning of the study (corresponding to postnatal day 70), were used to perform the experiments. Animals were single housed and kept in a reversed 12 h light/dark cycle (lights on at 8 p.m.) at constant temperature (20–22 °C) and humidity (45–55°), with food and water ad libitum. Animals were treated in accordance with the guidelines of the European Community Council Directive for Care and Use of Laboratory Animals (Ministry of Health Authorization n° 1D580.24) 

### 4.2. Drugs 

Nociceptin (Phe-Gly-Gly-Phe-Thr-Gly-Ala-Arg-Lys-Ser-Ala-Arg-Lys-Leu-Ala-Asp-Glu) (PM = 2379) and the NOP receptor antagonist UFP-101 ([Nphe(1),Arg(14),Lys(15)]N/OFQ NH(2)) (PM = 2706) were a generous gift of Prof. Remo Guerrini of the Department of Pharmaceutical Sciences, University of Ferrara, Italy. Both drugs were dissolved in sterile isotonic saline and injected icv in a volume of 1 µL/rat.

### 4.3. Surgical Procedures

MsP rats were anesthetized by intramuscular injection of 150 µL of tiletamine chlorohydrate (58.17 mg/10 mL) and zolazepam chlorohydrate (57.5 mg/10 mL) and placed into a stereotaxic frame. For the intracerebroventricular (icv) injections the skull was exposed and stainless steel guide cannula (diameter, 0.35 mm; length, 7 mm) was implanted with the following coordinates, anterior-posterior (AP), −1.0; lateral (L), −1.8; ventral (V), 2.0; [38]. The guide cannula were fixed to the skull with dental cement and two anchoring screws. After the surgery animals were allowed one week of recovery before starting the behavioural experiments. Drugs were administered via a 10 µL Hamilton syringe.

### 4.4. Experimental Procedures

#### 4.4.1. Chronic Intermittent EtOH Consumption in a Two Bottle Free-Choice Paradigm

Wistar and msP rats were trained to drink 10% alcohol (*w*/*v*) every other day. The 10% alcohol solution (*w*/*v*) was prepared daily by diluting 210 mL of 95% alcohol in 1800 mL of tap water. EtOH was dispensed through graduated burettes equipped with a metal dispenser, guaranteeing free access to the alcohol solution within 24 h. Daily consumption was monitored 24 h from exposure to the alcoholic solution. Rats were subjected to a chronic intermittent EtOH exposure (CIE) consisting of repeated cycles of one day in which they had free choice between a 10% alcohol solution and water for 24 h and the subsequent day in which they received the two burettes filled with only water. This intermittent exposure to alcohol continued for 30 days. The rats underwent 24 h ethanol withdrawal before sacrifice. To avoid the development of place preference, the position of alcohol or water (vehicle) containing burettes were alternated daily. One group of msP and one group of Wistar rats were used as controls and drunk only water for the entire experimental period. Animals had *ad libitum* access to food for the whole duration of the experiments.

#### 4.4.2. Intracerebroventricular Injection of N/OFQ and UFP-101 

After recovery from intracranial surgery, the msP rats were separated into three groups with similar body weight. Animals were given 1 μL of saline icv for 3 consecutive days to familiarize them with the injection procedure. Then, the first group was injected icv with isotonic saline (control), whereas the second and the third groups received 1 µg/µL/rat of N/OFQ [39,70] or 10 µg/µL/rat of UFP 101 [71,72], respectively. The stainless-steel injector protruding beyond the cannula tip by 2.5 mm was allowed to remain in the brain 1 min before being retracted. Animals of these three groups were sacrificed 4 h after microinjection for tissue collection. This time point was chosen because it corresponds to the time needed to detect changes in gene expression [73,74,75].

### 4.5. Tissue Collection

Twenty- four hours after the last EtOH exposure (day 31) rats were sacrificed and the brain areas of interest were removed and quickly frozen on dry ice. Brains were then placed onto an ice-cold matrix with 1 mm coronal section slice intervals. Tissue samples were taken by using Harris Uni-Core^TM^ punchers. The AMY was punched from −2 mm to −3 mm from bregma with a 1.5 mm diameter tip puncher. The BNST was collected from the slice taken 0 to −1 mm from bregma with a 1 mm puncher diameter (Figure 2). Brains were dissected under a stereomicroscope and the areas were collected in accordance with the rat brain atlas [38]. Tissues were stored at –80 °C until gene expression analysis.

### 4.6. Quantitative Real-Time RT-PCR

Total RNA was extracted according to the method of Chomczynski and Sacchi [76]. Each sample (*n* = 6 per group) was subjected to DNase treatment and converted to cDNA with the GeneAmp RNA PCR kit (Life Technologies Italia, Monza, Italy), according to the manufacturer’s protocol. Quantitative Real-time PCR analysis was performed on a StepOne Real-Time PCR System (Life Technologies) using the SYBR^®^ Green PCR MasterMix (Life Technologies); each sample was run in triplicate. Relative expression of different gene transcripts was calculated by the Delta-Delta Ct (DDCt) method and converted to relative expression ratio (2^−DDCt^) for statistical analysis [77]. All data were normalized to the housekeeping gene glyceraldehyde-3-phosphate dehydrogenase (GAPDH). The specificity of each PCR product was determined by melting curve analysis, constructed in the range of 60 °C to 95 °C [78]. Primers used for PCR amplification were designed using Primer 3, and are reported in Table 2.

### 4.7. Data Analysis

Behavioral data were initially evaluated by Shapiro-Wilk tests to confirm the normality of the distribution and by Levene tests for the homogeneity of variance. Once these properties have been confirmed the statistics was performed using the analysis of variance (ANOVA). Data from the two-bottle free choice experiment were evaluated by a mixed ANOVA with one factor within (time) and one factor between (strain) (*n* = 12). When appropriate, analyses were followed by Tukey’s multiple comparison tests. Biochemical data have been initially evaluated by Shapiro-Wilk tests to confirm the normality of the distribution and by Grubb’s test to identify outliers. Relative gene expression data were analyzed by two-way ANOVA followed by Sidak’s multiple comparison test tests (*n* = 5–6). The effect of the NOP agonist or antagonist on investigated mRNA levels were analyzed by means of a one-way ANOVA followed by Dunnett’s multiple comparison test (*n* = 5–6). For the statistical analysis, the GraphPad Prism 8 software, (San Diego, CA, USA) was used. Results are expressed as mean ± standard error of the mean (SEM). The level of significance was set at *p* < 0.05. 

## 5. Conclusions

The present results highlight how CIE drinking may result in a divergent regulation of stress-related neuropeptidergic systems, depending on the regions examined and the mechanisms involved. If in the AMY pro-stress dynorphin and CRF transmission are generally reduced following CIE, the opposite occurs in the BNST. Another important consideration is that alcohol elicited different gene expression changes in Wistar and in msP rats with the latter showing mostly higher fluctuations in response to alcohol. One possibility is that this different sensitivity could be linked to the distinct genetic background of the two lines. However, it should be also considered that msP rats, due to their innate high predisposition for alcohol intake, reached a much higher level of drinking, during CIE exposure. Hence, it is also possible that the differences in response to alcohol observed between the two rat lines depend on the amount of exposure to the substance. A clear finding emerged from this study is that msP rats show a different regulation of stress-related neuropeptidergic systems in the extended amygdala, compared to Wistar controls. This dysregulation may contribute to the expression of their innate high predisposition to drink alcohol and to their high anxiety and stress vulnerable phenotype. 

## Figures and Tables

**Table 1:** Summary table depicting the direction of gene expression changes detected in the AMY and the BNST of Wistar and mSP rats following water or CIE drinking. Increase (↑); decrease (↓); no changes (=).

Brain Area	Genotype/Treatment	Gene							
		pN/OFQ	NOP	pDYN	KOP	CRF	CRFR1	BDNF	TrkB
**AMY**	Wistar EtOH vs. *Wistar vehicle*	=	=	=	=	=	=	=	↑
	msP vehicle vs. *Wistar vehicle*	↑	↑	=	=	↑	=	=	↑
	msP EtOH vs. msP vehicle	↓	↓	↓	↓	↓	=	↑	=
**BNST**	Wistar EtOH vs. *Wistar vehicle*	=	=	=	=	↓	=	=	=
	msP vehicle vs. *Wistar vehicle*	↑	↑	=	=	↑	↑	↑	=
	msP EtOH vs. msP vehicle	↓	↑	=	↑	↑	↑	=	=

**Table 2:** Primer sequences used for real-time qPCR.

Gene	Forward (5′–3′)	Reverse (5′–3′)
*pN/OFQ*	TGCAGCACCTGAAGA GAATG	CAACTTCCGGGCTGACTTC
*NOP*	AGCTTCTGAAGAGGCTGTGT	GACCTCCCAGTATGGAGCAG
*CRF*	GCAGCGGGACTTCTGTTGA	CGCAGCCGTTGAATTTCTTG
*Pdyn*	CCTGTCCTTGTGTTCCCTGT	AGAGGCAGTCAGGGTGAGAA
*KOP*	TTGGCTACTGGCATCATCTG	ACACTCTTCAAGCGCAGGAT
*CRF1R*	TGCCAGGAGATTCTCAACGAA	AAAGCCGAGATGAGGTTCCAG
*BDNF*	AAGTCTGCATTACATTCCTCGA	GTTTTCTGAAAGAGGGACAGTTTAT
*TrkB*	AAGTTCTACGGTGTCTGTGTG	TTCTCTCCTACCAAGCAGTTC
*GAPDH*	AGACAGCCGCATCTTCTTGT	CTTGCCGTGGGTAGAGTCAT

## Data Availability

Data supporting the findings of this study are available upon reasonable request.
